# Jasplakinolide induces primary cilium formation through cell rounding and YAP inactivation

**DOI:** 10.1371/journal.pone.0183030

**Published:** 2017-08-10

**Authors:** Tomoaki Nagai, Kensaku Mizuno

**Affiliations:** Department of Biomolecular Sciences, Graduate School of Life Sciences, Tohoku University, Sendai, Japan; Institute of Molecular and Cell Biology, SINGAPORE

## Abstract

Primary cilia are non-motile cilia that serve as cellular antennae for sensing and transducing extracellular signals. In general, primary cilia are generated by cell quiescence signals. Recent studies have shown that manipulations to increase actin assembly suppress quiescence-induced ciliogenesis. To further examine the role of actin dynamics in ciliogenesis, we analyzed the effect of jasplakinolide (Jasp), a potent inducer of actin polymerization, on ciliogenesis. Unexpectedly, Jasp treatment induced ciliogenesis in serum-fed cells cultured at low density. In contrast, Jasp had no apparent effect on ciliogenesis in cells cultured at higher densities. Jasp-induced ciliogenesis was correlated with a change in cell morphology from a flat and adherent shape to a round and weakly adherent one. Jasp treatment also induced the phosphorylation and cytoplasmic localization of the YAP transcriptional co-activator and suppressed cell proliferation in low density-cultured cells. Overexpression of an active form of YAP suppressed Jasp-induced ciliogenesis. These results suggest that Jasp induces ciliogenesis through cell rounding and cytoplasmic localization and inactivation of YAP. Knockdown of LATS1/2 only faintly suppressed Jasp-induced YAP phosphorylation, indicating that LATS1/2 are not primarily responsible for Jasp-induced YAP phosphorylation. Furthermore, overexpression of active Src kinase suppressed Jasp-induced cytoplasmic localization of YAP and ciliogenesis, suggesting that down-regulation of Src activity is involved in Jasp-induced YAP inactivation and ciliogenesis. Our data suggest that actin polymerization does not suppress ciliogenesis *per se* but rather that cell rounding and reduced cell adhesion are more crucially involved in Jasp-induced ciliogenesis.

## Introduction

Primary cilia are microtubule-based sensory organelles that protrude from the plasma membranes of most vertebrate cells. They are non-motile cilia that serve as cellular antennae for sensing and transducing a variety of chemical and mechanical signals from the extracellular environment [1, 2]. Primary cilia play an essential role in the development and homeostasis of various tissues; therefore, defects in the formation or function of primary cilia cause diverse human diseases, collectively termed ciliopathies, including polycystic kidney disease, retinal degeneration, neurodevelopmental disorders, and situs inversus [1, 2]. The formation of primary cilia is tightly associated with cell cycle arrest or exit; in cultured cells, primary cilia are generated under the conditions that lead to cell quiescence, such as serum starvation and high confluence culture [3–5]. Primary cilia are formed through a multistep process that includes the formation of the ciliary vesicle at the distal end of the mother centriole, translocation of the mother centriole to the plasma membrane to form the basal body, extension of axonemal microtubules from the basal body, and transport of ciliary components into cilia [6, 7]. Previous studies have identified numerous factors that regulate the individual steps of ciliogenesis [6–9]; however, the mechanism by which cell quiescence signals induce primary cilium formation has remained elusive [3–5]. Recent studies have implicated changes in actin filament dynamics and organization in quiescence-induced ciliogenesis [9–11].

Actin filament dynamics and reorganization play essential roles in various cell activities, including migration, morphogenesis, division, and vesicular transport. Actin filament dynamics also play a crucial role in transducing the signals of cell geometry and mechanical states, connecting them to the gene expression that drives cell growth and proliferation [12, 13]. YAP (Yes-associated protein) is a transcriptional co-activator that promotes cell proliferation by associating with transcription factors, such as TEAD (TEA domain transcription factor) [14, 15]. YAP activity is negatively regulated by the Hippo pathway, a kinase cascade consisting of the MST1/2 and LATS1/2 kinases, which phosphorylate YAP and thereby promote its cytoplasmic retention or degradation [14, 15]. Several lines of evidence suggest that YAP is a key mediator in transducing cytoskeletal and mechanical signals into cell proliferation [16–25]. Under the conditions suitable for cell proliferation (such as serum feeding, low cell density, and stiff substrate), actin filaments tend to assemble, and YAP is translocated to the nucleus to stimulate cell proliferation; however, the inhibition of actin polymerization or Rho signaling results in cytoplasmic retention and inactivation of YAP and suppression of cell proliferation, even though cells are under growth-promoting conditions [16–25]. Inversely, under the conditions of cell quiescence (such as serum starvation, high cell density, and soft substrate), actin filaments tend to disassemble, and YAP is retained in the cytoplasm; however, forced stimulation of actin polymerization causes nuclear localization and activation of YAP and promotes cell proliferation, even under growth-inhibitory conditions [16–25]. Thus, it seems likely that actin assembly facilitates cell proliferation by promoting the nuclear translocation and activation of YAP, whereas actin disassembly leads to cell quiescence through the cytoplasmic retention and inactivation of YAP.

Because primary cilia are formed by cell quiescence signals and resorbed under cell proliferating signals, it may be presumed that actin disassembly promotes while actin assembly represses ciliogenesis by inhibiting and promoting cell cycle progression, respectively. In accordance with this assumption, several studies have shown that actin disassembly resulting from treatment with cytochalasin D (CytoD) or knockdown of the actin nucleator Arp3 promotes ciliogenesis, while actin assembly resulting from the knockdown of actin-severing factors, such as gelsolin or cofilin, represses ciliogenesis [9, 10]. However, the mechanisms by which actin dynamics regulate YAP activity and ciliogenesis remain largely unknown.

To further examine the role of actin dynamics in primary cilium formation, we analyzed the effect of jasplakinolide (Jasp), a potent inducer of actin polymerization, on ciliogenesis. In contrast to the model in which actin assembly represses ciliogenesis, we observed that Jasp treatment induced ciliogenesis under growth-promoting conditions. We provide evidence that Jasp induces ciliogenesis through cell rounding, Src inactivation, and YAP inactivation. Our results suggest that actin polymerization does not suppress ciliogenesis *per se* but rather that changes in cell shape and adhesiveness are more crucially involved in Jasp-induced primary cilium formation.

## Materials and methods

### Antibodies, plasmids, siRNAs, and reagents

Monoclonal antibodies against acetyl (Ac)-tubulin (Sigma), β-actin (Sigma), YAP (Santa Cruz Biotechnology), and Myc-tag (Medical & Biological Laboratories), and polyclonal antibodies against Arl13b (Proteintech), GFP (Molecular Probes), LATS1 (Cell Signaling), and LATS2 (Bethyl Laboratories) were purchased from the indicated suppliers. Alexa-488-labeled anti-Ki-67 antibody and Alexa-633-labeled phalloidin were purchased from Abcam and Thermo Fisher Scientific, respectively. The cDNA for human YAP was cloned by PCR amplification. The plasmid encoding YAP(5SA), in which five serine residues (S61, S109, S127, S164, and S397) were replaced by alanine [26], was constructed with a site-directed mutagenesis kit (Agilent). The cDNA plasmids encoding wild-type (WT) LIMK1 and its kinase-dead (KD) mutant, in which Asp-460 was replaced by Ala, were constructed as described previously [27]. The cDNA plasmids encoding (Myc+His)-tagged Src and its constitutively active (ΔC, amino acids 1–522) and KD (with a replacement of Asp-389 by Ala) mutants were constructed, as described previously [28, 29]. The siRNAs targeting LATS1, LATS2, MST1, MST2, NDR1, NDR2, and TTBK2 were purchased from Thermo Fisher Scientific. The siRNA targeting sequences were as follows; 5’-CCU CCA UAC GAG UCA AUC A-3’ (LATS1 siRNA #1), 5’-GGA GUG AUG AUA ACG AGG A-3’ (LATS1 siRNA #2), 5’-GUU CGG ACC UUA UCA GAA A-3’ (LATS2 siRNA #1), 5’-GCA UUU UAC GAA UUC ACC U-3’ (LATS2 siRNA #2), 5’-GGA UGG AGA CUA CGA GUU U-3’ (MST1 siRNA), 5’-GAG AUA CAC UGC GAA AAG A-3’ (MST2 siRNA), 5’-GCA AUG AAA AUA CUC CGU A-3’ (NDR1 siRNA), 5’-GGU UUG AAG GGU UGA CUC A-3’ (NDR2 siRNA), and 5’-GUC AUG ACA UGU UAC CCA A-3’ (TTBK2 siRNA). Jasplakinolide (Jasp, Adipogen), latrunculin B (LatB, Sigma), and cytochalasin D (CytoD, Sigma) were purchased from the indicated suppliers.

### Cell culture and transfection

Human telomerase reverse transcriptase (hTERT)-immortalized retinal pigmented epithelial (RPE)-1 cells (hereafter referred to as RPE1 cells) were provided by H. Nakanishi (Kumamoto University, Japan) and M. Matsuyama (Okayama University, Japan). RPE1 cells were cultured in Dulbecco’s modified Eagle’s medium (DMEM)/Ham’s F-12 (Wako Pure Chemical) supplemented with 10% fetal calf serum (FCS). Transfections with plasmids and siRNAs were performed using Lipofectamine LTX with PLUS and Lipofectamine RNAiMAX (Thermo Fisher Scientific), respectively. To examine the effects of Jasp on ciliogenesis, RPE1 cells were plated on coverslips in 6-well culture plates at low (2.1 x 10^3^ cells/cm^2^), medium (1.0 x 10^4^ cells/cm^2^), or high (5.2 x 10^4^ cells/cm^2^) densities. Cells were usually treated with 0.5 μM Jasp for 24 h in 10% FCS-containing medium and then fixed. For overexpression or knockdown experiments, RPE1 cells were transfected with plasmids or siRNAs, cultured for 24 h, and then further cultured for 24 h at low density in 10% serum-containing medium in the presence of 0.5 μM Jasp or control DMSO before fixation. To examine the effect of EDTA treatment on ciliogenesis, RPE1 cells were cultured on poly-L-lysine-coated coverslips in 6-well culture plates at low density. Cells were treated with 6 mM EDTA for 24 h in 10% FCS-containing medium and then fixed.

### Immunostaining and fluorescence microscopy

Immunostaining was carried out as described previously [30]. Cells were fixed with 4% paraformaldehyde in phosphate-buffered saline (PBS), followed by permeabilization with 0.1% Triton X-100 in PBS. Cells were stained with primary antibody diluted with Can-Get-Signal immunostain (Toyobo) and secondary antibody diluted with 2% FCS in PBS. Fluorescence images were obtained using a DMI6000B fluorescence microscope (Leica Microsystems) equipped with a PL Apo 63x oil objective lens and CCD camera (Cool SNAP HQ, Roper Scientific).

### Immunoblotting

Immunoblotting was carried out as described previously [31]. To analyze the level of YAP phosphorylation, cells were lysed with pre-heated sodium dodecyl sulfate (SDS)-containing lysis buffer (1% SDS, 50 mM Tris-HCl pH 7.5, and 1 mM dithiothreitol) and boiled for 20 min at 100°C. Cell lysates were analyzed by SDS-polyacrylamide gel electrophoresis (PAGE) using SDS-polyacrylamide gels containing 20 μM Phos-tag (Wako Pure Chemical), according to the manufacturer’s instructions. Proteins were transferred to polyvinylidene difluoride (PVDF) membranes using Mini Trans-Blot Electrophoretic Transfer Cells (Bio-Rad) according to the manufacturer’s instructions. Protein-transferred membranes were subjected to immunoblot analyses using a standard protocol.

### F-actin sedimentation assay

F-actin sedimentation assay was carried out as described previously [32]. Briefly, Jasp-treated RPE1 cells were lysed in lysis buffer (50 mM HEPES pH 7.4, 100 mM NaCl, 1 mM MgCl_2_, 0.2 mM CaCl_2_, 1 mM dithiothreitol, 0.2 mM ATP, 1% NP-40, and 2 μM phalloidin). Cell lysates were centrifuged at 100,000 xg for 30 min at 4°C. Equal amounts of pellet and supernatant were subjected to SDS-PAGE and analyzed by immunoblotting with an anti-β-actin antibody.

### Statistical analyses

Data are expressed as means ± SEM of three to four independent experiments. All statistical analyses were carried out by Prism 6 (GraphPad Software). *P*-values were calculated using unpaired Student’s *t*-tests for two-group comparisons and one-way ANOVA followed by Dunnett’s test for multiple data set comparisons. In all cases, *P* < 0.05 was considered statistically significant.

## Results

### Jasp treatment induces ciliogenesis

**Fig 1:**
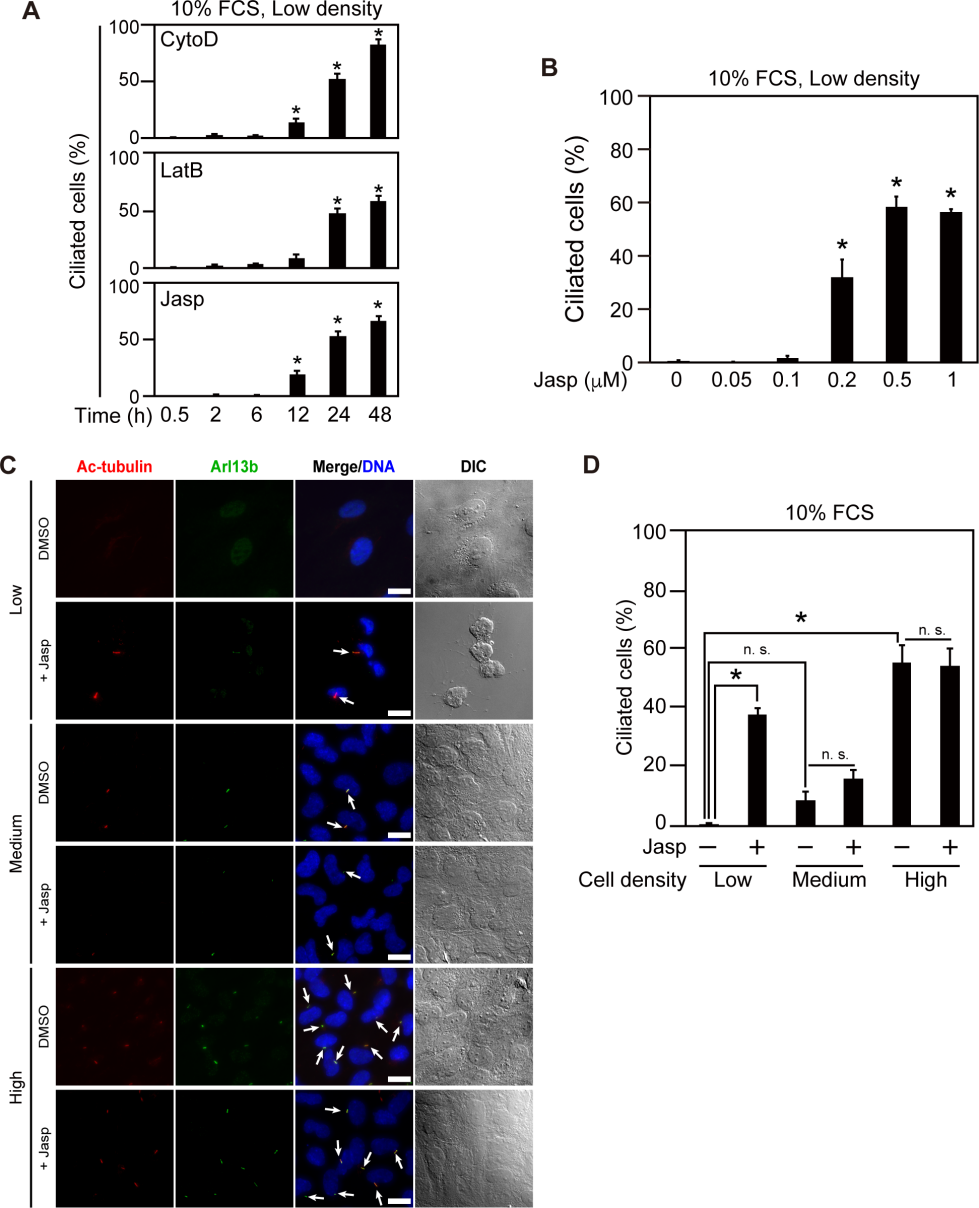
Jasplakinolide (Jasp) induces ciliogenesis and cell rounding in cells cultured at low density. (A) Effects of actin-modulating drugs on ciliogenesis. RPE1 cells were cultured at low density in serum-containing medium; treated with 0.5 μM CytoD, 1 μM LatB, or 1 μM Jasp for the indicated lengths of time; and then fixed. The percentage of ciliated cells was counted based on staining for Ac-tubulin and Arl13b. (B) Dose-dependent effect of Jasp on ciliogenesis. RPE1 cells were cultured at low density in serum-containing medium, treated with the indicated concentrations of Jasp for 24 h, and then fixed. The percentage of ciliated cells was analyzed as in (A). (C) Effects of Jasp on ciliogenesis and cell morphology in cells cultured at distinct cell densities. RPE1 cells were cultured at low (2.1 x 10^3^ cells/cm^2^), medium (1.0 x 10^4^ cells /cm^2^), or high (5.2 x 10^4^ cells /cm^2^) density in serum-containing medium, treated with 0.5 μM Jasp for 24 h, and then fixed. Cells were stained with anti-Ac-tubulin (red) and anti-Arl13b (green) antibodies. DNA was stained with DAPI (blue). Arrows indicate primary cilia. Scale bar, 20 μm. (D) Quantification of the frequency of ciliated cells in Jasp-treated or untreated cells cultured at distinct densities. In (A), (B), and (D), data are means ± SEM from three independent experiments. *P < 0.05; n.s., not significant.

Previous studies showed that treatment with CytoD or knockdown of Arp3 promotes ciliogenesis, whereas knockdown of gelsolin or cofilin represses ciliogenesis [9, 10]. These results suggest that actin disassembly promotes and actin assembly represses ciliogenesis. To further examine the roles of actin dynamics in ciliogenesis, we analyzed the effects of various drugs that induce actin assembly or disassembly on ciliogenesis. RPE1 cells were cultured at low cell density (2.1 x 10^3^ cells/cm^2^) in 10% serum-supplemented medium (i.e., under growth-promoting conditions that usually suppress cilium formation), treated with actin-modulating drugs for 0.5–48 h, and then fixed and stained with antibodies against Ac-tubulin and Arl13b to detect the formation of primary cilia. As reported [9, 10], treatment with CytoD or LatB, drugs that inhibit actin polymerization, increased the frequency of ciliated cells in a time-dependent manner (Fig 1A). Unexpectedly, treatment with Jasp, a drug that induces actin polymerization [33], also increased the frequency of ciliated cells in a time- and concentration-dependent manner (Fig 1A, 1B and 1C, first and second rows). F-actin sedimentation assays using ultracentrifugation confirmed that treatment with Jasp potently increased the ratio of F-actin to total actin in RPE1 cells (S1 Fig). These results indicate that Jasp treatment induces primary cilium formation in serum-fed cells cultured at low density, even though Jasp promotes actin polymerization.

### Jasp-induced ciliogenesis is correlated with cell rounding

We next examined whether Jasp induces ciliogenesis in cells cultured at higher cell densities. RPE1 cells were plated at low (2.1 x 10^3^ cells/cm^2^), medium (subconfluent, 1.0 x 10^4^ cells /cm^2^), or high (confluent, 5.2 x 10^4^ cells/cm^2^) density, cultured in 10% serum-containing medium, and treated with Jasp for 24 h; cilia were then analyzed by anti-Ac-tubulin and anti-Arl13b staining. As described above, when cells were cultured at low density, primary cilium formation was not observed in the absence of Jasp but was induced in 37% of Jasp-treated cells (Fig 1C and 1D). When cultured at medium and high densities in the absence of Jasp, primary cilia were observed in 8% and 55% of cells, respectively, even in the presence of serum (Fig 1C and 1D). In contrast to cells at low density, Jasp treatment had no significant effect on ciliogenesis in cells at medium or high density (Fig 1C and 1D). Notably, Jasp-induced ciliogenesis was correlated with a change in cell morphology; differential interference contrast (DIC) microscopy showed that most cells at low density exhibited a dramatic change in morphology from a flat and adherent shape to a round and weakly adherent one upon Jasp treatment; in contrast, cells cultured at medium or high density retained the flat and adherent shape even after Jasp treatment (Fig 1C, DIC images). These observations suggest that cell rounding and reduced cell attachment to the dish are involved in Jasp-induced ciliogenesis in low density-cultured cells.

### Jasp treatment induces cell quiescence and YAP cytoplasmic localization and phosphorylation

**Fig 2:**
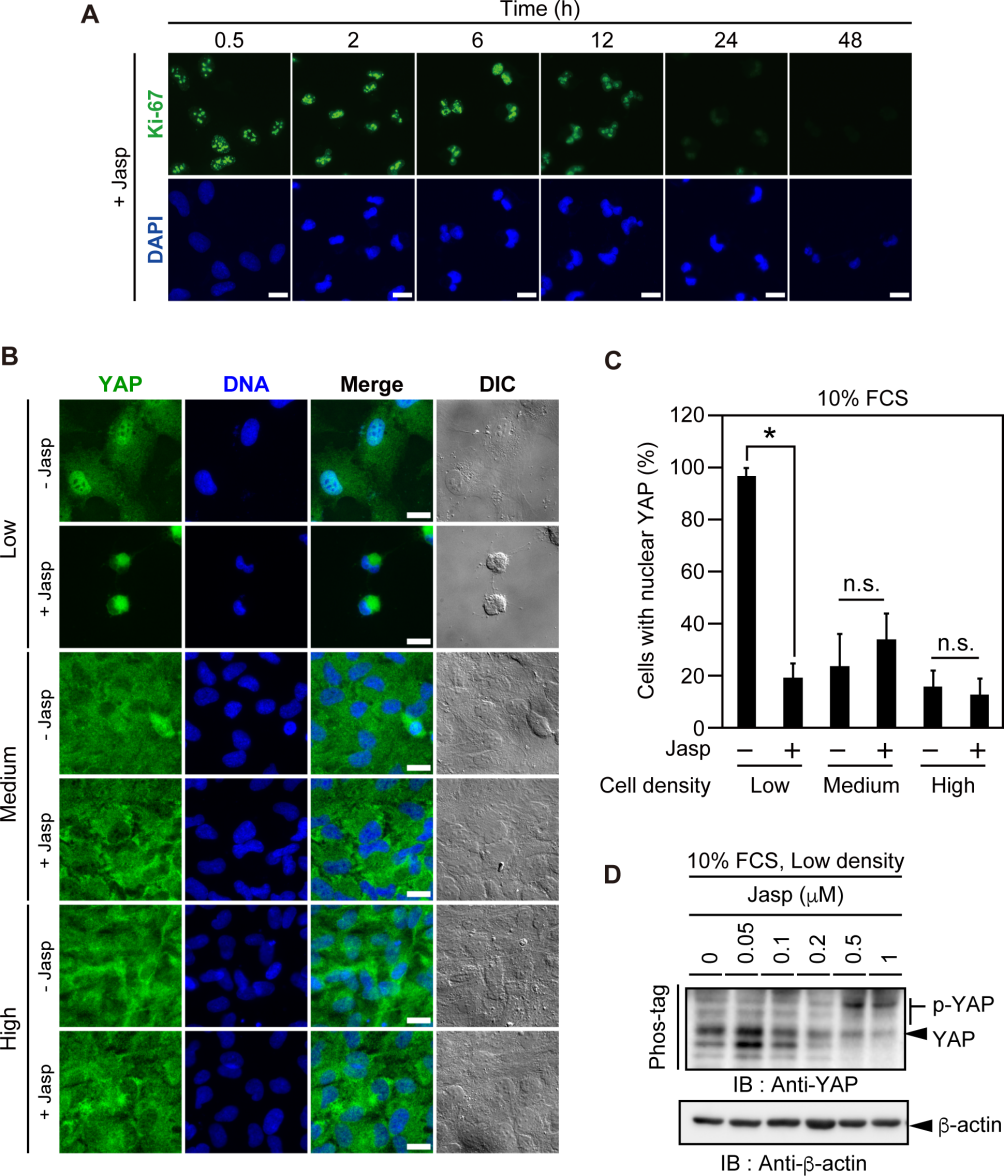
Jasp treatment induces cell quiescence and the cytoplasmic localization and phosphorylation of YAP. (A) Effect of Jasp treatment on cell proliferation. RPE1 cells were cultured at low density in serum-containing medium, treated with 1 μM Jasp for the indicated lengths of time, and then fixed and stained with Alexa-488-conjugated anti-Ki-67 antibody (green). DNA was stained with DAPI (blue). Scale bar, 20 μm. (B) Jasp induces the cytoplasmic localization of YAP in cells at low density. RPE1 cells were cultured at low, medium, and high densities in serum-containing medium, treated with 0.5 μM Jasp for 24 h, and then fixed and stained with anti-YAP antibody (green). DNA was stained with DAPI (blue). DIC images are shown in the right panels. Scale bar, 20 μm. (C) Quantification of the effects of Jasp treatment on YAP localization. The percentage of cells with YAP localization in the nucleus (preferentially in the nucleus or equally in the nucleus and cytoplasm) was counted. Data are means ± SEM from three independent experiments. *P < 0.05; n.s., not significant. (D) Jasp promotes YAP phosphorylation. RPE1 cells were cultured at low density in serum-containing medium and treated with the indicated concentrations of Jasp for 24 h. Cell lysates were subjected to Phos-tag-containing and normal SDS-PAGE and analyzed by immunoblotting with anti-YAP and anti-β-actin antibodies, respectively.

Primary cilia are generally formed under cell quiescence conditions. We next asked whether Jasp treatment induces cell quiescence. RPE1 cells cultured at low density in serum-containing medium were treated with Jasp for 0.5–48 h and then fixed and stained for Ki-67, a commonly used marker for proliferating cells. Most cells were Ki-67-positive before Jasp treatment, but the number of Ki-67-positive cells markedly decreased 24–48 h after treatment with Jasp (Fig 2A), indicating that Jasp treatment induces cell quiescence. The time course of Jasp-induced cell quiescence was similar to that of ciliogenesis, indicating that Jasp-induced ciliogenesis is tightly associated with cell quiescence.

YAP is a transcriptional co-activator promoting cell proliferation. YAP activity is inhibited by phosphorylation and cytoplasmic retention [14, 15]. Previous studies showed that changes in cell morphology and actin dynamics regulate YAP localization and phosphorylation [16–25] and that CytoD-induced actin disassembly suppresses YAP activity and thereby promotes ciliogenesis [10]. Since Jasp treatment induces cell rounding, quiescence, and ciliogenesis, we next examined whether Jasp affects the subcellular localization and phosphorylation of YAP. RPE1 cells cultured at low, medium, or high density in serum-containing medium were treated with Jasp for 24 h and stained with an anti-YAP antibody. At low cell density, YAP mostly localized to the nucleus in untreated control cells, but Jasp treatment caused cell rounding and translocation of YAP to the cytoplasm (Fig 2B). Quantitative analysis showed that more than 90% of cells exhibited YAP localization in the nucleus in the absence of Jasp but that Jasp treatment markedly reduced the number of cells with nuclear YAP localization, with more than 80% of Jasp-treated cells exhibiting YAP localization predominantly in the cytoplasm (Fig 2C). At medium and high densities, YAP predominantly localized to the cytoplasm in most cells in the absence of Jasp, and Jasp treatment had no significant effect on cell morphology or YAP localization (Fig 2B and 2C). Immunoblot analysis after Phos-tag-containing SDS-PAGE revealed that levels of phosphorylated YAP (p-YAP) in cells at low density markedly increased upon treatment with Jasp at concentrations of 0.5–1.0 μM (Fig 2D). These results indicate that Jasp treatment causes YAP inactivation by promoting its phosphorylation and cytoplasmic localization.

A previous study showed that intraperitoneal injection of high concentrations of Jasp causes nuclear translocation and activation of YAP in high-density cells in zebrafish blastemal region [34]. So, we further examined the effects of higher concentrations of Jasp on YAP localization and ciliogenesis in high-density RPE1 cells. In the absence of Jasp, about 90% of RPE1 cells cultured at high density exhibited YAP localization predominantly in the cytoplasm (S2 Fig) and about 70% of cells formed cilia (S3 Fig). Treatment of these cells with Jasp at concentrations of 0.2–5.0 μM had no significant effect on the cytoplasmic localization of YAP (S2 Fig) and the ratio of ciliated cells (S3 Fig). Treatment with 2–5 μM Jasp caused cell rounding, but such treatment had no further effect on YAP localization and ciliogenesis (S2 and S3 Figs). Thus, in contrast to the results obtained in zebrafish in vivo models, treatment with high concentrations of Jasp did not promote nuclear localization of YAP in RPE1 cells cultured at high density.

### Overexpression of active YAP suppresses Jasp-induced ciliogenesis

**Fig 3:**
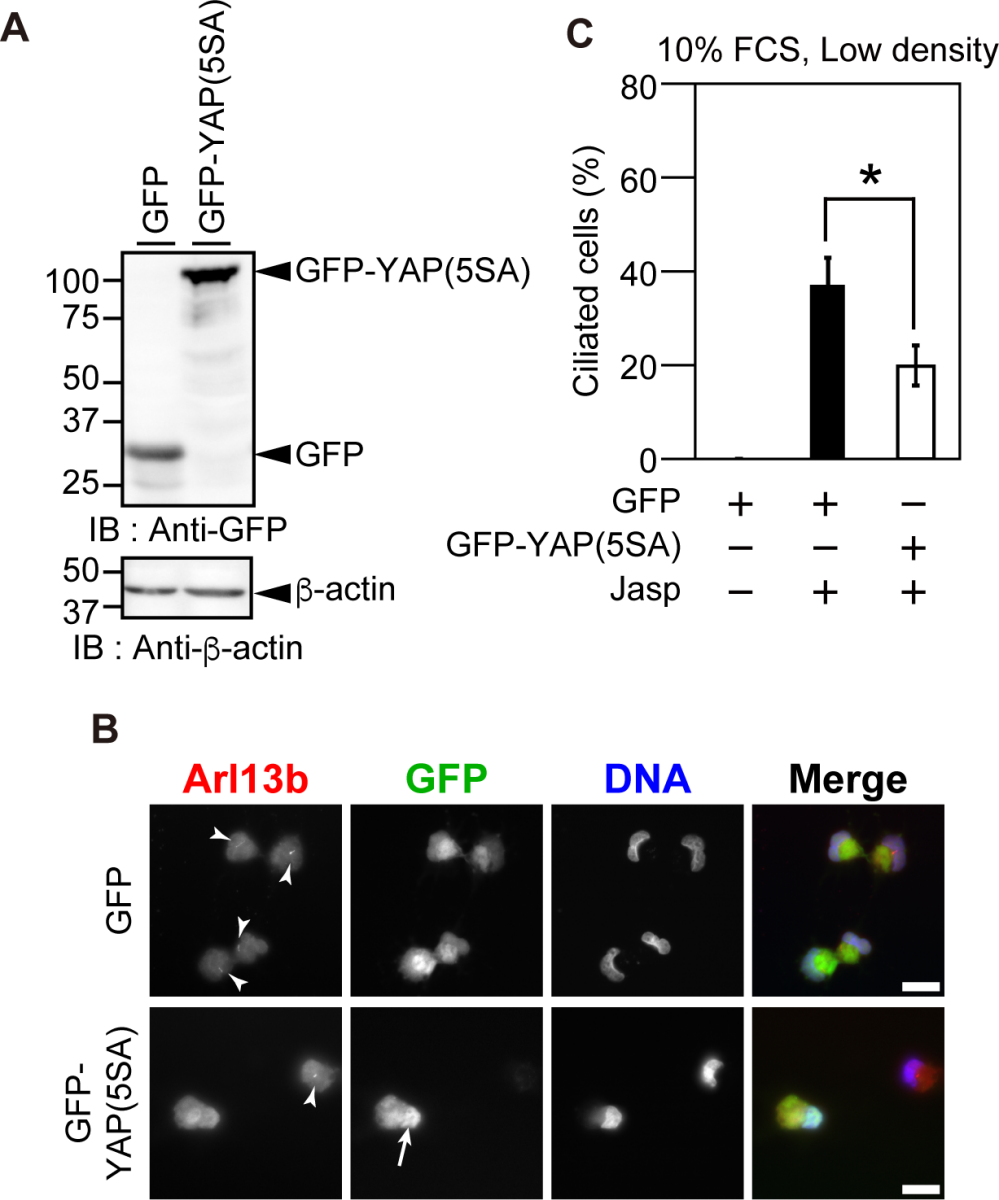
Overexpression of active YAP suppresses Jasp-induced ciliogenesis. (A) Expression of GFP or GFP-YAP(5SA) in Jasp-treated RPE1 cells. RPE1 cells transfected with GFP or GFP-YAP(5SA) were cultured at low density in serum-containing medium and treated with 0.5 μM Jasp for 24 h. Cell lysates were subjected to SDS-PAGE and analyzed by immunoblotting with anti-GFP and anti-β-actin antibodies. (B) Effect of YAP(5SA) overexpression on Jasp-induced ciliogenesis. RPE1 cells were transfected with GFP or GFP-YAP(5SA) and treated with Jasp as in (A), and then fixed and stained with anti-Arl13b antibody (red) and DAPI (blue). Cells were also imaged by GFP fluorescence (green). Arrowheads indicate primary cilia. Arrow indicates nuclear localization of GFP-YAP(5SA). Scale bar, 20 μm. (C) Quantification of the percentage of ciliated cells among GFP-positive cells. Data are means ± SEM from four independent experiments. *P < 0.05.

Recent studies showed that YAP activation suppresses ciliogenesis [10]. Since Jasp treatment was found to cause YAP inactivation, we next examined the role of YAP inactivation in Jasp-induced ciliogenesis. To do this, we constructed a constitutively active form of YAP, YAP(5SA), in which five serine residues involved in phosphorylation and inactivation of YAP were substituted with alanine [26]. RPE1 cells were transfected with GFP-tagged YAP(5SA) or control GFP, cultured at low density in serum-containing medium, and treated with Jasp for 24 h. Immunoblotting with anti-GFP antibody confirmed the expression of GFP or GFP-YAP(5SA) (Fig 3A). Immunofluorescence analyses revealed that GFP-YAP(5SA) mostly localized to the nucleus, even after Jasp treatment (Fig 3B), indicating that YAP(5SA) is resistant to Jasp-induced cytoplasmic translocation and inactivation. Jasp treatment induced cilium formation in control GFP-expressing cells, but expression of GFP-YAP(5SA) significantly suppressed Jasp-induced ciliogenesis (Fig 3B and 3C). These results suggest that YAP inactivation is involved in Jasp-induced ciliogenesis.

### Effects of LATS1/2 knockdown on Jasp-induced YAP phosphorylation and ciliogenesis

**Fig 4:**
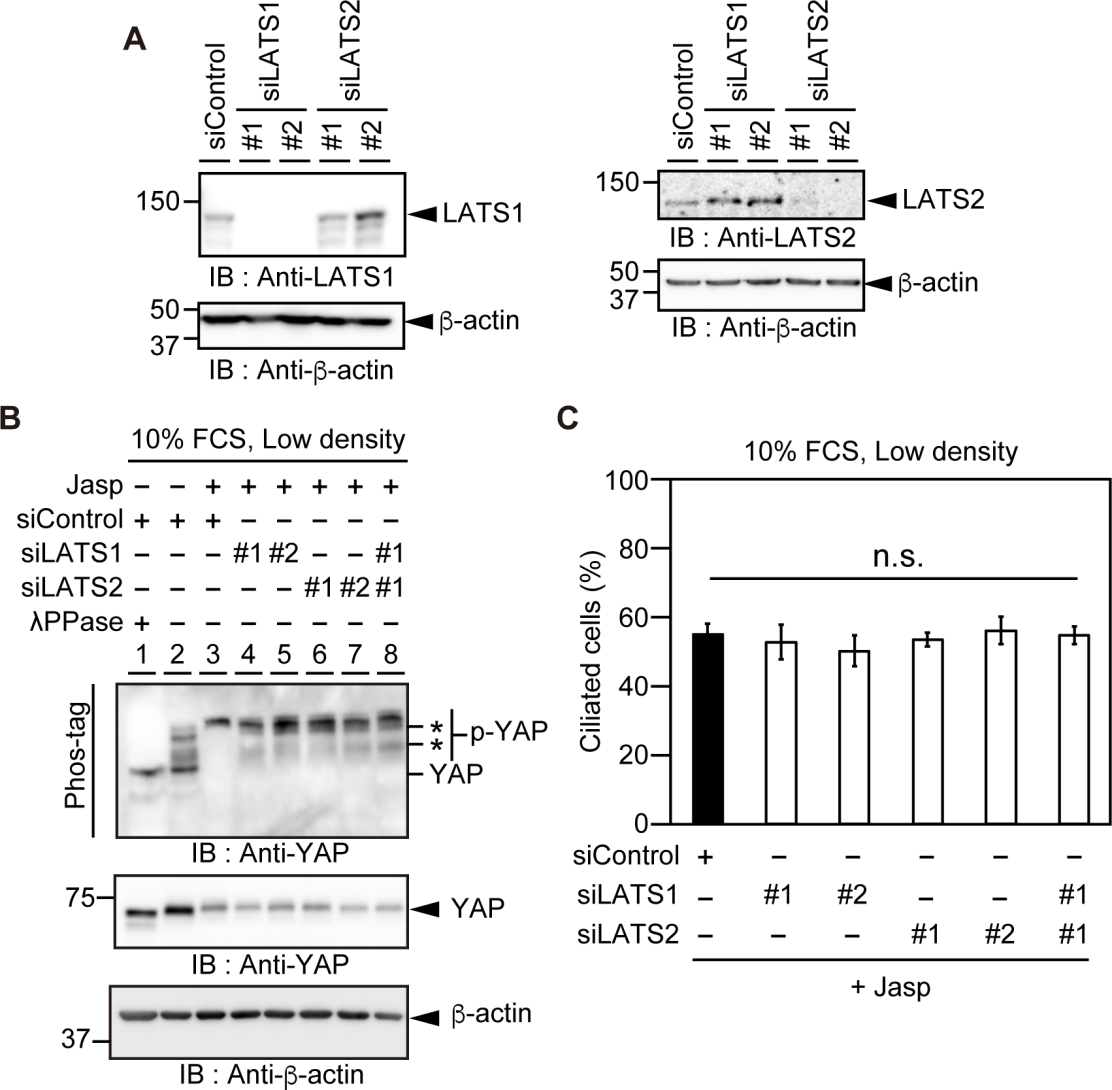
Effects of LATS1/2 knockdown on Jasp-induced YAP phosphorylation and ciliogenesis. (A) Effects of LATS1/2 siRNAs on the expression of LATS1 (left) and LATS2 (right). RPE1 cells were transfected with control, LATS1, or LATS2 siRNAs and cultured for 48 h. Cell lysates were analyzed by immunoblotting with antibodies against LATS1, LATS2, and β-actin. (B) Effects of LATS1/2 knockdown on Jasp-induced YAP phosphorylation. RPE1 cells were transfected with control siRNA or LATS1/2 siRNAs, cultured for 24 h at low density in serum-containing medium, and then further incubated with 0.5 μM Jasp for 24 h. Cell lysates were subjected to Phos-tag-containing or conventional SDS-PAGE and analyzed by immunoblotting with anti-YAP and anti-β-actin antibodies. In lane 1, lysates were treated with λ phosphatase. Asterisks indicate the positions of partially dephosphorylated YAP. (C) Effects of LATS1/2 knockdown on Jasp-induced ciliogenesis. RPE1 cells were transfected with control siRNA or LATS1/2 siRNAs, cultured for 24 h at low density in serum-containing medium, further incubated with 0.5 μM Jasp for 24 h, and then fixed. The percentage of ciliated cells was counted based on staining for Ac-tubulin and Arl13b. Data are means ± SEM from four independent experiments. n.s., not significant.

LATS1 and LATS2 are protein kinases that phosphorylate and inactivate YAP [14, 15]. To examine the role of LATS1/2 in Jasp-induced YAP phosphorylation and ciliogenesis, we analyzed the effects of LATS1/2 knockdown. The siRNAs targeting LATS1 and LATS2 effectively reduced the expression of each target protein in RPE1 cells (Fig 4A). RPE1 cells transfected with LATS1/2 siRNAs were cultured at low density in serum-containing medium and treated with Jasp for 24 h; levels of YAP phosphorylation were then analyzed by gel mobility shift assay using Phos-tag-containing SDS-PAGE. In control siRNA-treated cells, Jasp treatment caused the gel mobility shift of YAP (Fig 4B, lanes 2 and 3), indicating that Jasp treatment markedly increases the level of YAP phosphorylation. In cells with single or double knockdown of LATS1/2, YAP-immunoreactive bands with higher gel mobility (indicated by asterisks) were detected (Fig 4B, lanes 4–8), indicating that single or double knockdown of LATS1/2 slightly suppresses Jasp-induced YAP phosphorylation. In addition, the total level of YAP seemed to decrease upon Jasp treatment (lanes 3–8, middle panel in Fig 4B), which may reflect the proteasome-dependent degradation of phosphorylated YAP [35, 36]. We also examined the effects of LATS1/2 knockdown on ciliogenesis by counting the frequency of ciliated cells. Single or double knockdown of LATS1/2 had no apparent effect on Jasp-induced ciliogenesis compared with control siRNA treatment (Fig 4C). These results indicate that LATS1/2 are only partially involved in Jasp-induced YAP phosphorylation and that they are dispensable for Jasp-induced ciliogenesis. It seems likely that knockdown of LATS1/2 is not sufficient to increase YAP activity to the level required for inhibiting ciliogenesis. Thus, LATS1/2 appear to play only a minor role in Jasp-induced YAP inactivation and ciliogenesis.

We also analyzed the effects of the knockdown of MST1/2 or NDR1/2, other kinase components of the Hippo pathway [14, 37–40], on Jasp-induced ciliogenesis. Knockdown of MST1/2 or NDR1/2 had no significant effect on Jasp-induced ciliogenesis (S4 Fig). In contrast, the knockdown of TTBK2, a protein kinase essential for ciliogenesis [30, 41, 42], strongly suppressed Jasp-induced ciliogenesis (S4 Fig).

### Overexpression of Src suppresses Jasp-induced ciliogenesis and cytoplasmic translocation of YAP

**Fig 5:**
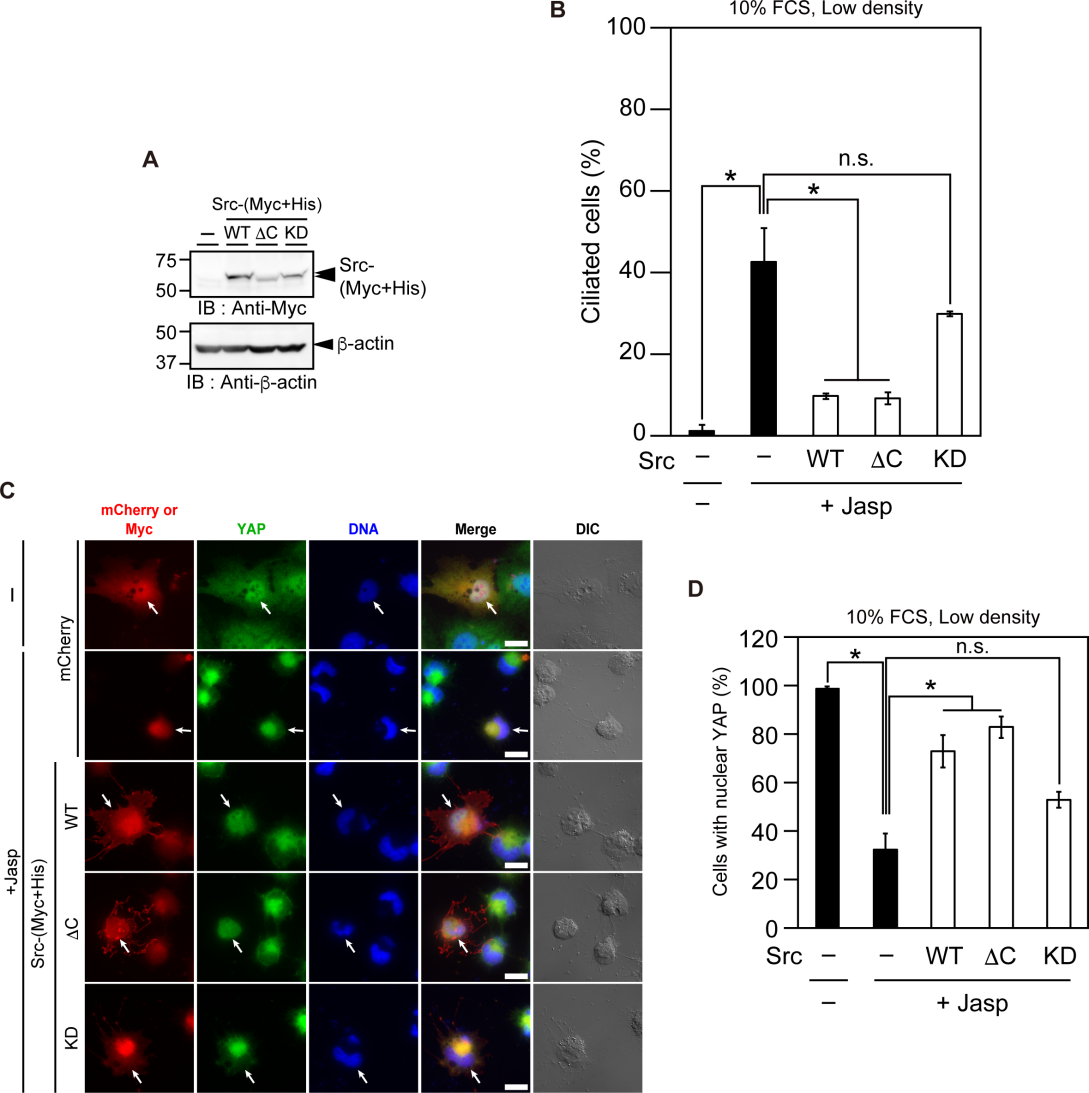
Overexpression of Src suppresses Jasp-induced ciliogenesis and the cytoplasmic translocation of YAP. (A) Expression of Src-(Myc+His) or its mutants in Jasp-treated RPE1 cells. RPE1 cells were transfected with (Myc+His)-tagged Src(WT), or its active (ΔC) or kinase-dead (KD) mutant; cultured at low density in serum-containing medium; treated with 0.5 μM Jasp for 24 h. Cell lysates were subjected to SDS-PAGE and analyzed by immunoblotting with anti-Myc and anti-β-actin antibodies. (B) Effect of Src overexpression on Jasp-induced ciliogenesis. RPE1 cells were transfected with (Myc+His)-tagged Src or its mutants or control mCherry, cultured, and treated with Jasp, as in (A), and then fixed. The percentage of ciliated cells was counted based on staining for Ac-tubulin and Arl13b. (C) Effect of Src overexpression on Jasp-induced cytoplasmic translocation of YAP. RPE1 cells were transfected, cultured, treated with Jasp, and then fixed, as in (B). Cells were stained with anti-Myc (red) and anti-YAP (green) antibodies. In the first and second rows, cells were imaged by mCherry fluorescence (red). DNA was stained with DAPI (blue). DIC images are shown in the right panels. Arrows indicate the mCherry- or Myc-positive cells. Scale bar, 20 μm. (D) Quantification of the effect of Src overexpression on the localization of YAP. The percentage of cells with nuclear localization of YAP was analyzed, as in Fig 2C. In (B) and (D), data are means ± SEM from three independent experiments. *P < 0.05; n.s., not significant.

As mentioned above, Jasp treatment caused cell rounding and decreased cell attachment to the dish. Src is a tyrosine kinase that is activated downstream of integrin-mediated cell adhesion and inactivated after cell detachment from the substratum [43]. To examine the role of cell rounding and detachment in Jasp-induced ciliogenesis, we analyzed the effect of Src overexpression on Jasp-induced ciliogenesis. RPE1 cells were transfected with (Myc+His)-tagged Src(WT) or its mutants, cultured at low density in serum-fed medium, and treated with Jasp for 24 h. Immunoblotting with anti-Myc antibody confirmed the expression of Src-(Myc+His) or its mutants in Jasp-treated RPE1 cells (Fig 5A). Expression of Src(WT) or its constitutively active mutant, ΔC, significantly suppressed Jasp-induced cilium formation, compared to that in control mCherry-expressing cells (Fig 5B). In contrast, expression of a kinase-dead (KD) mutant of Src had no significant effect on Jasp-induced ciliogenesis (Fig 5B). These results suggest that Src kinase activity has an inhibitory role in Jasp-induced ciliogenesis and that Src inactivation is involved in Jasp-induced ciliogenesis.

We also examined the effect of Src overexpression on Jasp-induced cytoplasmic localization of YAP. RPE1 cells were transfected with Src(WT) or its mutants, cultured at low density in serum-containing medium, and treated with Jasp for 24 h. Overexpression of Src(WT) and its constitutively active ΔC mutant significantly blocked Jasp-induced cytoplasmic localization of YAP, but expression of the KD form of Src had no significant effect (Fig 5C and 5D). These results suggest that Src activation blocks Jasp-induced YAP inactivation and that Jasp induces the cytoplasmic localization and inactivation of YAP through Src inactivation.

### Overexpression of LIMK1 suppresses serum starvation-induced ciliogenesis in adherent cells

**Fig 6:**
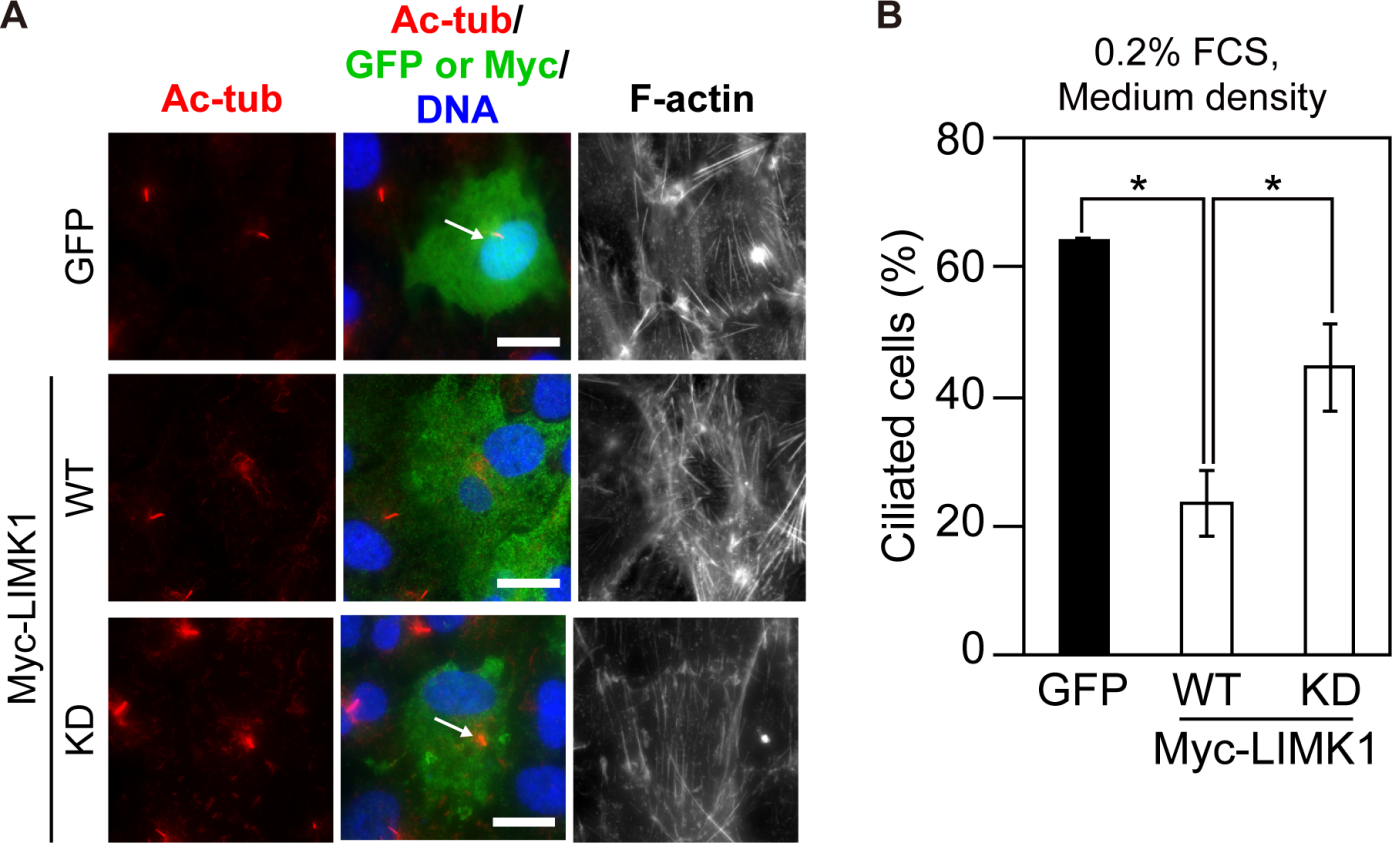
Overexpression of LIMK1 suppresses serum starvation-induced ciliogenesis in adherent cells. (A) Effect of LIMK1 overexpression on serum starvation-induced ciliogenesis. RPE1 cells were transfected with GFP or Myc-tagged wild-type (WT) or kinase-dead (KD) LIMK1, cultured at medium density in serum-fed medium, and then subjected to serum starvation for 48 h. Cells were fixed and stained with anti-Ac-tubulin (red) and anti-Myc (green) antibodies and DAPI (blue). Cells were also imaged by GFP fluorescence (green). Actin filaments were stained with Alexa-633-phalloidin (right panels). Arrows indicate primary cilia. Scale bar, 20 μm. (B) Quantification of the frequency of ciliated cells. The percentage of ciliated cells was counted among GFP- or Myc-positive cells, based on staining for Ac-tubulin. Data are means ± SEM from three independent experiments. *P < 0.05.

Above, we demonstrated that Jasp induces cell rounding and ciliogenesis in RPE1 cells cultured at low density. However, several studies have shown that actin polymerization suppresses serum starvation-induced ciliogenesis in adherent cells [9, 10, 44, 45]. To examine whether actin polymerization actually suppresses serum starvation-induced ciliogenesis under our experimental conditions, RPE1 cells were transfected with Myc-tagged LIMK1, a protein kinase that phosphorylates and inactivates cofilin and thereby stimulates actin polymerization [27, 32]; cells were then cultured at medium density in serum-starved medium for 48 h. Expression of LIMK1(WT) significantly suppressed serum starvation-induced ciliogenesis, but its KD mutant did not (Fig 6), suggesting that LIMK1-induced actin polymerization indeed suppresses serum starvation-induced ciliogenesis. Similar results were reported with the expression of other cofilin kinases, LIMK2 and TESK1 [10]. These results are apparently inconsistent with the ciliogenesis-promoting effect of Jasp in low density-cultured cells (Fig 1). However, unlike Jasp treatment, overexpression of LIMK1(WT) did not cause cell rounding. Thus, although both Jasp and LIMK1 induce actin polymerization, they have distinct effects on ciliogenesis, probably due to their different effects on cell morphology and adhesiveness and actin cytoskeletal organization.

### EDTA-induced cell rounding does not induce ciliogenesis

As described above, we showed that Jasp-induced ciliogenesis is associated with cell rounding and YAP inactivation. To examine whether cell rounding and subsequent YAP inactivation are sufficient for ciliogenesis, we analyzed whether EDTA-mediated cell rounding would induce ciliogenesis in RPE1 cells. Treatment with EDTA caused cell rounding and detachment (DIC images in S5A Fig). Immunostaining of YAP revealed that similar to Jasp treatment, EDTA treatment caused cytoplasmic localization of YAP (S5A and S5B Fig). However, in contrast to Jasp treatment, EDTA treatment did not induce ciliogenesis (S5A and S5C Fig). These results suggest that cell rounding and YAP inactivation are not sufficient for triggering ciliogenesis.

## Discussion

**Fig 7:**
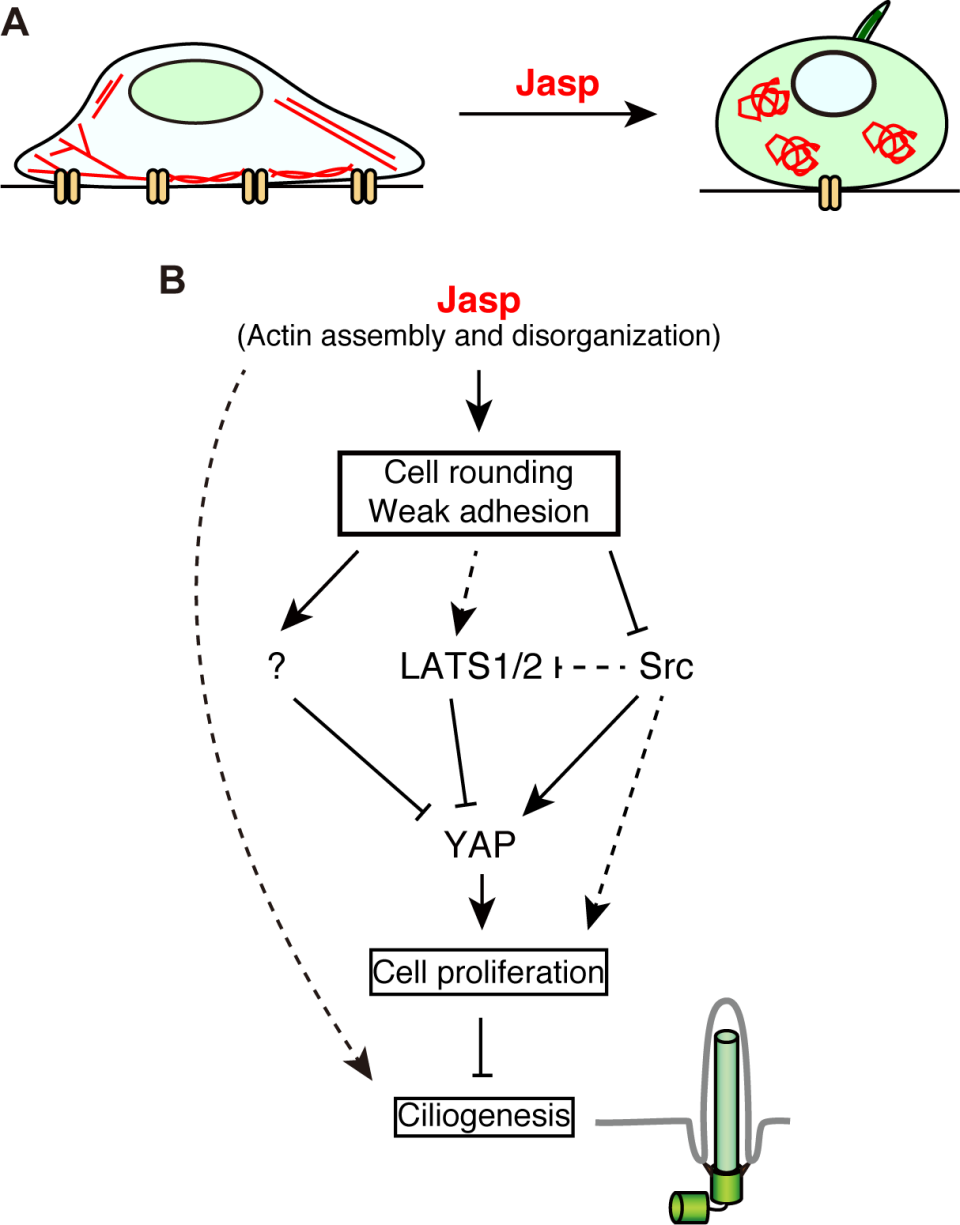
A model for Jasp-induced ciliogenesis. (A) Illustration of Jasp-induced changes in cell morphology and adhesiveness. (Left) When cells are cultured at low density in serum-containing medium, cells tightly adhere to the dish, actin filaments assemble into stress fibers, and YAP predominantly localizes to the nucleus. (Right) Upon Jasp treatment, cells become round and weakly adhere to the dish, actin filaments further assemble into disorganized structures, YAP translocates to the cytoplasm, and the primary cilium is formed. (B) A proposed signaling pathway of Jasp-induced ciliogenesis. Jasp-induced cell rounding and reduced adhesion cause YAP inactivation through the inactivation of Src and the activation of LATS1/2 and other unidentified protein kinases. YAP inactivation leads to cell quiescence and thereby causes ciliogenesis. As EDTA-induced cell rounding and YAP inactivation do not cause ciliogenesis, other pathway(s) independent of YAP inactivation seem to play a role in Jasp-induced ciliogenesis.

In this study, we showed that treatment with Jasp (at 0.5 μM) induces ciliogenesis in RPE1 cells cultured at low density and in serum-containing medium. Under these conditions, Jasp also induced a change in cell morphology from a flat and adherent shape to a round and weakly adherent one, the phosphorylation and cytoplasmic translocation of YAP, and cell quiescence. In contrast, in RPE1 cells cultured at medium or high density, treatment with 0.5 μM Jasp had no apparent effect on cell shape, YAP localization, or the frequency of ciliated cells. Cell rounding and reduced cell adhesion correlated well with YAP inactivation and ciliogenesis, suggesting that these changes in cell morphology and adhesiveness play a crucial role in Jasp-induced YAP inactivation and ciliogenesis. Overexpression of active Src markedly suppressed the Jasp-induced cytoplasmic translocation of YAP and ciliogenesis, further supporting the crucial role of the decline in cell–substrate adhesion in Jasp-induced YAP inactivation and ciliogenesis. Moreover, the overexpression of active YAP significantly suppressed Jasp-induced ciliogenesis, indicating the pivotal role of YAP inactivation in Jasp-induced ciliogenesis. YAP inactivation likely promotes ciliogenesis by suppressing cell proliferation and the transcription of the mitotic kinases, Plk1 and aurora-A, which are known to inhibit ciliogenesis [10, 46, 47]. Taken together, these results suggest that Jasp induces ciliogenesis in low density-cultured cells primarily through pathways involving cell rounding, the inhibition of Src activity, the cytoplasmic localization and inactivation of YAP, and cell quiescence (Fig 7).

To examine whether cell rounding is sufficient for ciliogenesis, we analyzed the effect of EDTA-mediated cell rounding on ciliogenesis. Treatment with EDTA caused cell rounding and cytoplasmic translocation of YAP, but did not induce ciliogenesis, which suggests that cell rounding and subsequent YAP inactivation are not sufficient for triggering ciliogenesis. A previous study showed that ciliogenesis requires not only YAP inactivation but also other mechanisms such as stimulation of vesicular transport to the ciliary base [10]. Thus, it is likely that Jasp treatment induces ciliogenesis by stimulating any unknown mechanisms that are required for ciliogenesis but are not induced by EDTA treatment, in addition to stimulating the pathway of cell rounding and YAP inactivation (Fig 7). Alternatively, it is also possible to consider that EDTA treatment may inhibit any mechanisms required for ciliogenesis.

Previous studies showed that treatments that induce actin assembly, such as knockdown of cofilin or gelsolin, suppress primary cilium formation [7, 9, 10]. We also showed that the overexpression of LIMK1 suppresses serum starvation-induced ciliogenesis. Several mechanisms by which actin assembly may inhibit ciliogenesis have been proposed, including the inhibition of vesicle transport required for cilium formation by branched actin networks [9–11, 44, 45] and the blocking of centrosome migration to the plasma membrane by stress fibers [48–50]. Actin fibers also reduce cilium length by recruiting histone deacetylase-6 to cilia, which leads to axoneme destabilization and represses cilium formation [51]. In addition, actin assembly and actomyosin-based mechanical tension appear to inhibit ciliogenesis indirectly by promoting YAP activation and cell proliferation, although the mechanisms by which cytoskeletal and mechanical signals regulate YAP activity are largely unknown [10, 16–25]. In contrast to these observations, we showed that treatment with Jasp leads to YAP inactivation and ciliogenesis, even though it promotes actin polymerization. Considering that Jasp induces ciliogenesis in cells cultured at low density but not at medium or high density and that the cell-rounding phenotype correlates well with ciliogenesis, it seems likely that Jasp induces ciliogenesis primarily through changes in cell morphology and adhesiveness. In addition, Jasp induces the accumulation of disorganized F-actin aggregates [33, 52, 53], whereas the knockdown of cofilin or gelsolin or overexpression of LIMK1 induces organized F-actin structures, such as stress fibers and branched F-actin networks. Such differences in actin cytoskeletal organization may also explain why Jasp-induced F-actin assembly has no inhibitory effect on ciliogenesis. Since the integrity of actin cytoskeleton is required for the nuclear localization of YAP [25], disruption of the actin cytoskeletal integrity may play a role in Jasp-induced cytoplasmic localization of YAP and ciliogenesis.

We showed that Jasp treatment induces cytoplasmic localization of YAP in RPE1 cells cultured at low density. In contrast, a recent study reported that intraperitoneal injection of the high concentration of Jasp causes nuclear translocation and activation of YAP in high-density cells in zebrafish blastema [34]. It remains unclear how Jasp induces distinct effects on YAP localization in these cells. The high concentration of Jasp causes cell rounding in 2D-cultured RPE1 cells even at high density, but does not cause such cell shape changes under in vivo 3D conditions. These differences in cell morphology may explain the distinct effects of Jasp on YAP localization in 2D-cultured RPE1 cells and in zebrafish blastema cells in in vivo 3D systems.

Cell adhesion to the substrate plays an important role in cell survival and proliferation. Cell attachment promotes YAP activation and cell proliferation, whereas cell detachment inhibits them [19, 54]. Since Jasp treatment weakens cell attachment, it is likely that cell detachment-induced YAP inactivation is involved in Jasp-induced ciliogenesis. It has been shown that cell detachment leads to the phosphorylation and cytoplasmic localization of YAP through the activation of LATS1/2 [54]. However, in our experimental system, knockdown of LATS1/2 only slightly suppressed Jasp-induced YAP phosphorylation and had no apparent effect on Jasp-induced ciliogenesis, indicating that LATS1/2 only slightly, if at all, contribute to Jasp-induced phosphorylation and inactivation of YAP and subsequent ciliogenesis. Similarly, previous studies also showed that LATS1/2 are dispensable for serum-starvation- or actin-disassembly-induced YAP phosphorylation and ciliogenesis [10, 17, 20, 23, 39]. These results suggest that other protein kinase(s) are crucially involved in Jasp- and actin-disassembly-induced YAP phosphorylation and inactivation. Identification of the responsible kinase(s) is important for understanding the mechanism of Jasp- and actin-disassembly-induced YAP phosphorylation/inactivation and ciliogenesis.

Src tyrosine kinase is activated downstream of integrin signaling at cell–substrate adhesion sites [43]. We showed that overexpression of active Src suppresses Jasp-induced cytoplasmic translocation of YAP and ciliogenesis, suggesting that a decrease in Src kinase activity is involved in Jasp-induced YAP inactivation and ciliogenesis. A previous study showed that the inactivation of Src promotes ciliogenesis by inhibiting cortactin-mediated actin polymerization [44]. However, it is unlikely that the inhibition of cortactin-mediated actin polymerization is involved in Jasp-induced ciliogenesis, as actin is highly polymerized in Jasp-treated cells. It was also reported that the inhibition of Src promotes the cytoplasmic localization of YAP by promoting LATS1/2 activity [55]. However, LATS1/2 play only minor roles in Jasp-induced YAP inactivation in our experimental system. More recently, Src was shown to directly phosphorylate YAP and thereby stimulate its nuclear localization and activation, which suggests that Src inactivation leads to YAP dephosphorylation [56]. However, Jasp treatment led to increased levels of YAP phosphorylation. Therefore, the mechanism by which Src inactivation is involved in Jasp-induced YAP inactivation and ciliogenesis remains unclear. Further studies are required to understand the molecular mechanisms by which Jasp-induced cell rounding and Src inactivation lead to YAP inactivation and ciliogenesis.

Collectively, we showed that Jasp treatment induces ciliogenesis in cells cultured at low density. We obtained evidence that Jasp induces ciliogenesis via a pathway consisting of cell rounding, Src inactivation, YAP cytoplasmic localization and inactivation, and cell quiescence. Our data suggest that Jasp-induced actin assembly is not involved in ciliogenesis and that changes in cell shape and adhesiveness are more critical in contributing to the regulation of YAP activity and ciliogenesis. Further studies are required to understand the precise mechanisms by which cell shape and adhesiveness regulate YAP activity and ciliogenesis.

## Supporting information

S1 Fig Effect of Jasp treatment on actin polymerization. (A) F-actin sedimentation assays. RPE1 cells were plated at low density in serum-containing medium and treated with the indicated concentrations of Jasp for 12 h. Cell lysates were ultracentrifuged, and the amounts of actin recovered in the supernatant (S) and pellet (P) were analyzed by immunoblotting with anti-β-actin antibody. (B) Quantification of the ratio of F-actin (P) to total actin (S+P). Data are means ± SEM from three independent experiments. *P < 0.05.
(TIF)

S2 Fig Effect of Jasp treatment on YAP localization in RPE1 cells at high density. (A) Dose-dependent effect of Jasp on YAP localization in RPE1 cells at high density. RPE1 cells were cultured at high density in serum-containing medium, treated with the indicated concentrations of Jasp for 24 h, and then fixed and stained with anti-YAP antibody (green). DNA was stained with DAPI (blue). DIC images are shown in the right panels. Scale bar, 20 μm. (B) Quantification of the effects of Jasp treatment on YAP localization. The percentage of cells with YAP localization in the nucleus was counted as in Fig 2C. Data are means ± SEM from three independent experiments. n.s., not significant.
(TIF)

S3 Fig Effect of Jasp treatment on ciliogenesis in RPE1 cells at high density. (A) Dose-dependent effect of Jasp on ciliogenesis in RPE1 cells at high density. RPE1 cells were cultured at high density in serum-containing medium, treated with the indicated concentrations of Jasp for 24 h, and then fixed. Cells were stained with anti-Ac-tubulin (red) and anti-Arl13b (green) antibodies. DNA was stained with DAPI (blue). DIC images are shown in the right panels. Arrows indicate primary cilia. Scale bar, 20 μm. (B) Quantification of the frequency of ciliated cells. The percentage of ciliated cells was counted based on staining for Ac-tubulin and Arl13b, as shown in (A). Data are means ± SEM from three independent experiments. n.s., not significant.
(TIF)

S4 Fig Effects of knockdown of MST1/2, NDR1/2, or TTBK2 on Jasp-induced ciliogenesis. RPE1 cells were transfected with control siRNA or siRNAs targeting MST1, MST2, NDR1, NDR2, or TTBK2, as indicated; cultured at low density in serum-containing medium for 24 h; and then treated with 0.5 μM Jasp for 24 h. The percentage of ciliated cells was counted based on staining for Ac-tubulin and Arl13b. Data are means ± SEM from three independent experiments. *P < 0.05; n.s., not significant.
(TIF)

S5 Fig Effects of EDTA treatment on cell shape, YAP localization, and ciliogenesis. (A) EDTA treatment induces cell rounding and YAP translocation to the cytoplasm. RPE1 cells were cultured at low density; treated with 6 mM EDTA for 24 h; fixed and stained with anti-Arl13b (red) and anti-YAP (green) antibodies. DNA was stained with DAPI. DIC images are shown in the right panels. Scale bars, 20 μm. (B) Quantification of the effect of EDTA treatment on YAP localization. The percentage of cells with YAP localization in the nucleus was counted as in Fig 2C. (C) Quantification of the effect of EDTA treatment on ciliogenesis. The percentage of ciliated cells was counted based on staining of Arl13b, as shown in (A). In (B) and (C), data are means ± SEM from three independent experiments. n.s., not significant.
(TIF)
