# Supplementary figures and images for: Jasplakinolide induces primary cilium formation through cell rounding and YAP inactivation
Source: PLoS One. 2017 Aug 10;12(8):e0183030. doi: 10.1371/journal.pone.0183030 (PMC5552318; doi:10.1371/journal.pone.0183030)

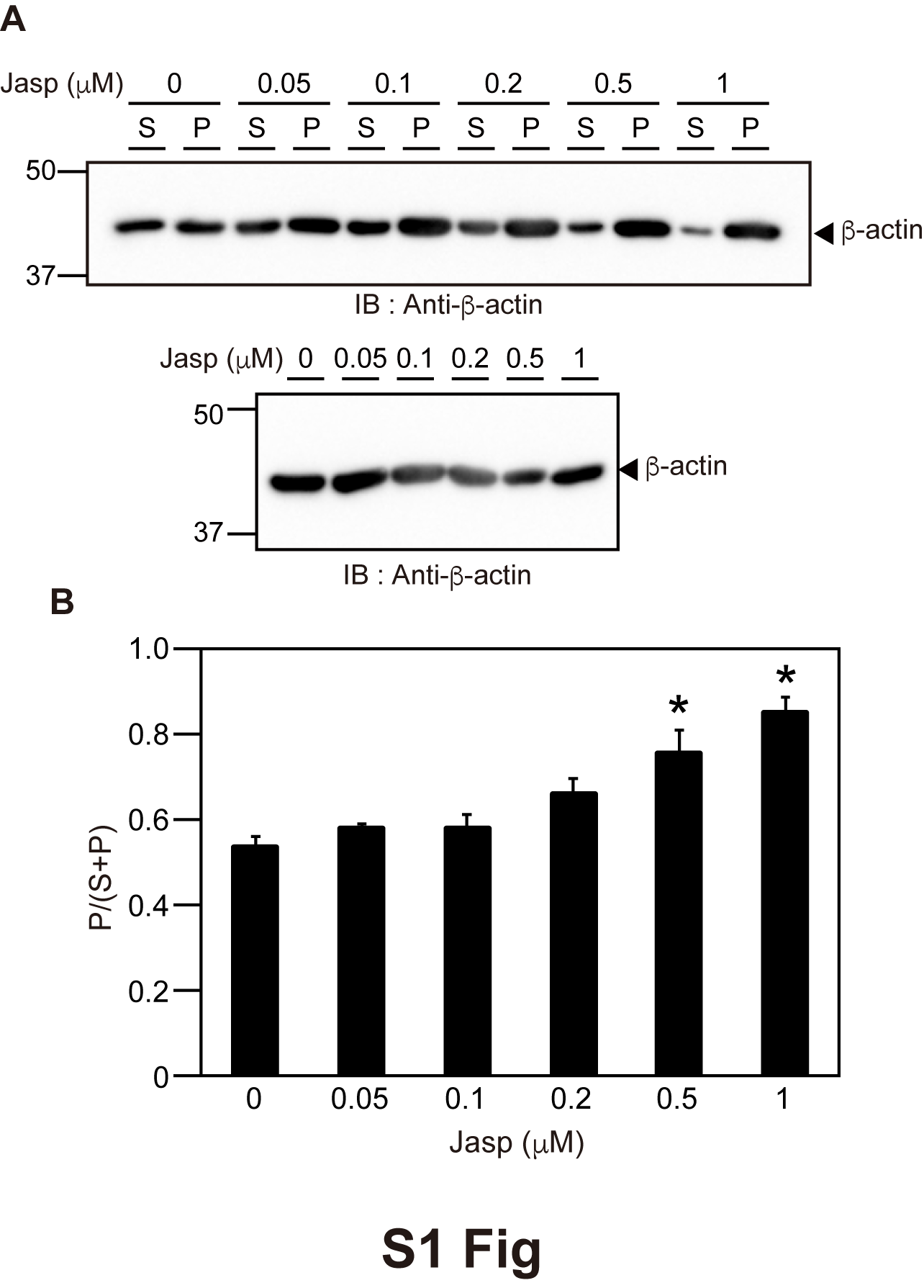

Supplement: S1 Fig — (A) F-actin sedimentation assays. RPE1 cells were plated at low density in serum-containing medium and treated with the indicated concentrations of Jasp for 12 h. Cell lysates were ultracentrifuged, and the amounts of actin recovered in the supernatant (S) and pellet (P) were analyzed by immunoblotting with anti-β-actin antibody. (B) Quantification of the ratio of F-actin (P) to total actin (S+P). Data are means ± SEM from three independent experiments. *P < 0.05. (TIF) [file pone.0183030.s001.tif]

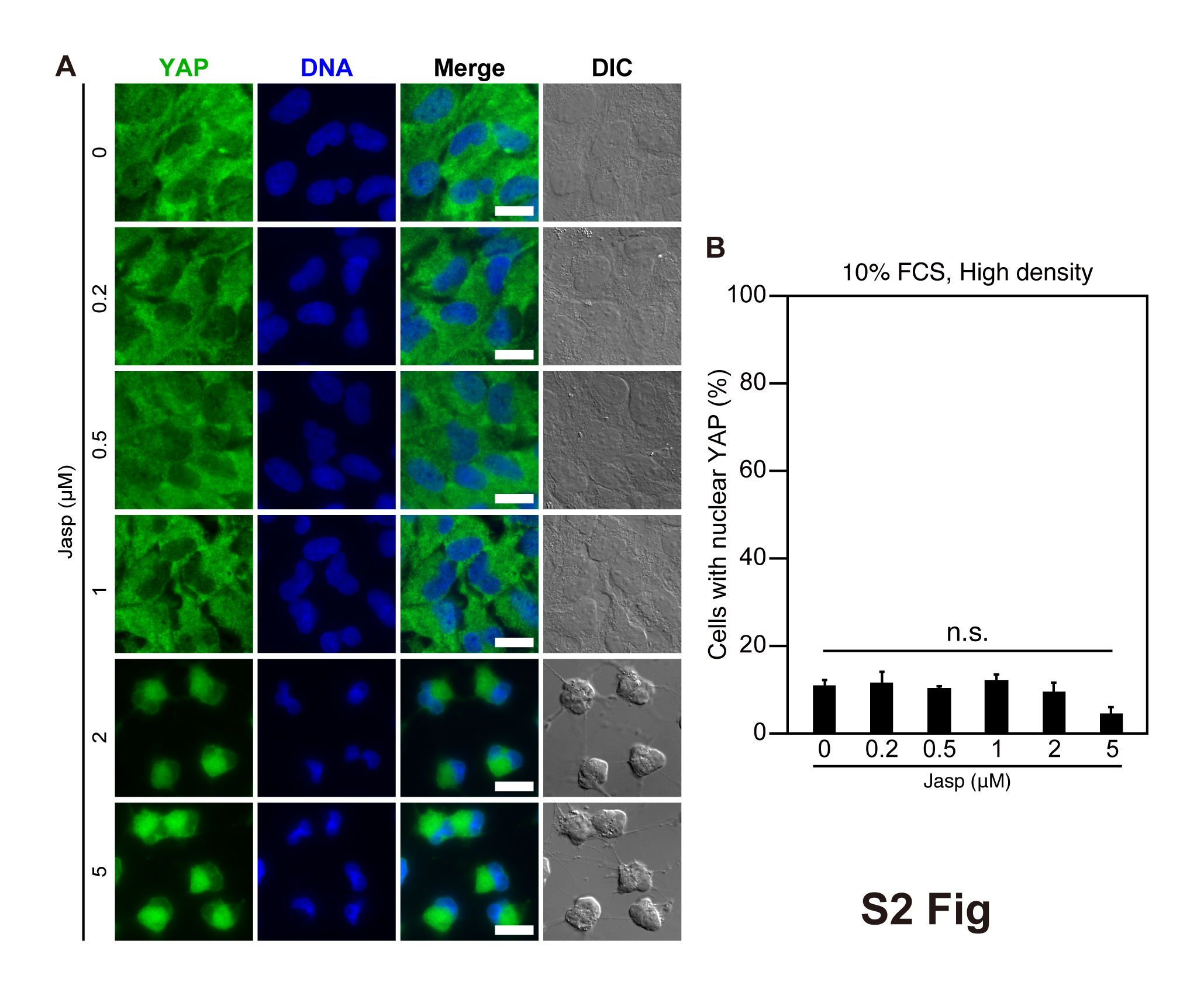

Supplement: S2 Fig — (A) Dose-dependent effect of Jasp on YAP localization in RPE1 cells at high density. RPE1 cells were cultured at high density in serum-containing medium, treated with the indicated concentrations of Jasp for 24 h, and then fixed and stained with anti-YAP antibody (green). DNA was stained with DAPI (blue). DIC images are shown in the right panels. Scale bar, 20 μm. (B) Quantification of the effects of Jasp treatment on YAP localization. The percentage of cells with YAP localization in the nucleus was counted as in Fig 2C. Data are means ± SEM from three independent experiments. n.s., not significant. (TIF) [file pone.0183030.s002.tif]

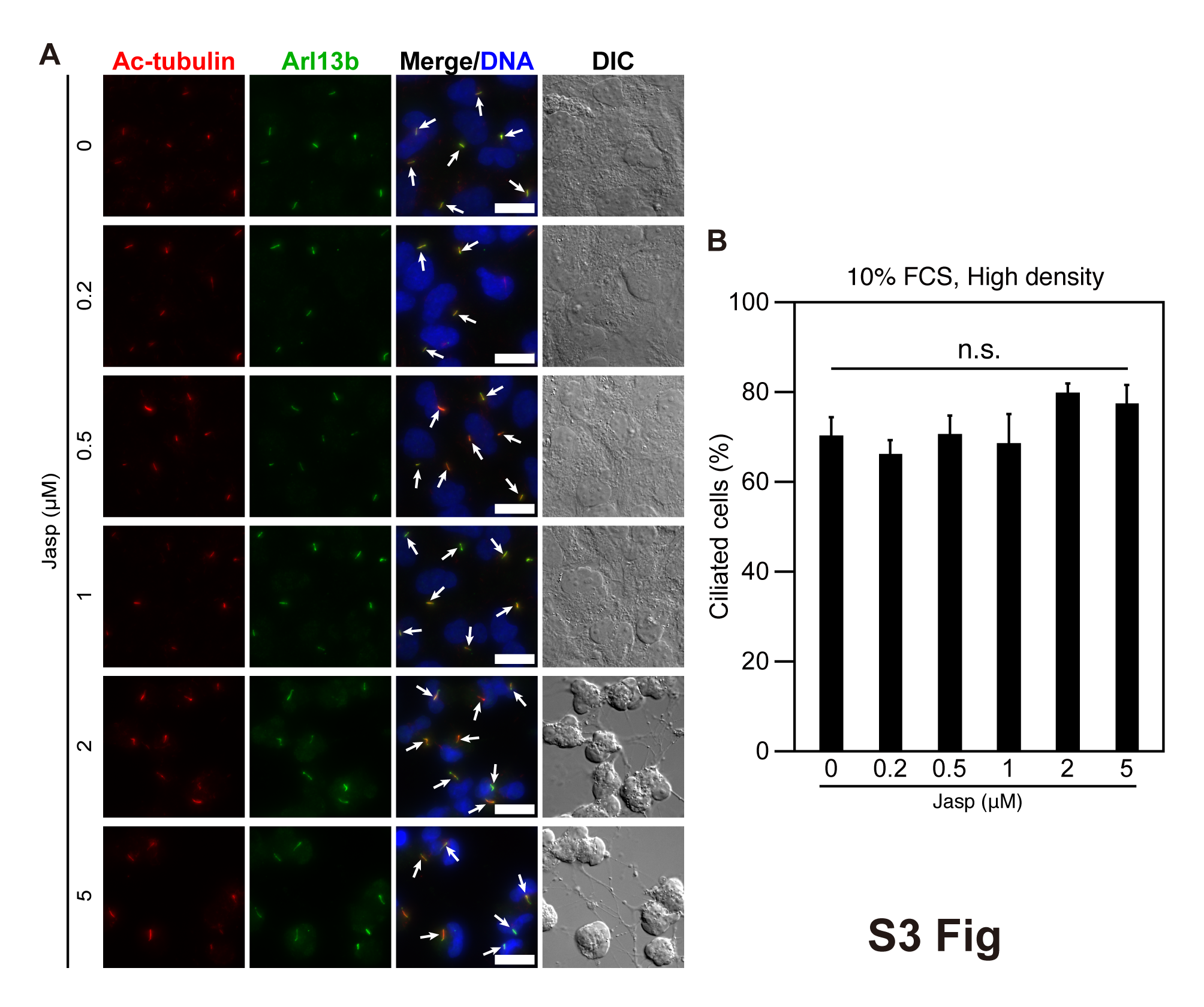

Supplement: S3 Fig — (A) Dose-dependent effect of Jasp on ciliogenesis in RPE1 cells at high density. RPE1 cells were cultured at high density in serum-containing medium, treated with the indicated concentrations of Jasp for 24 h, and then fixed. Cells were stained with anti-Ac-tubulin (red) and anti-Arl13b (green) antibodies. DNA was stained with DAPI (blue). DIC images are shown in the right panels. Arrows indicate primary cilia. Scale bar, 20 μm. (B) Quantification of the frequency of ciliated cells. The percentage of ciliated cells was counted based on staining for Ac-tubulin and Arl13b, as shown in (A). Data are means ± SEM from three independent experiments. n.s., not significant. (TIF) [file pone.0183030.s003.tif]

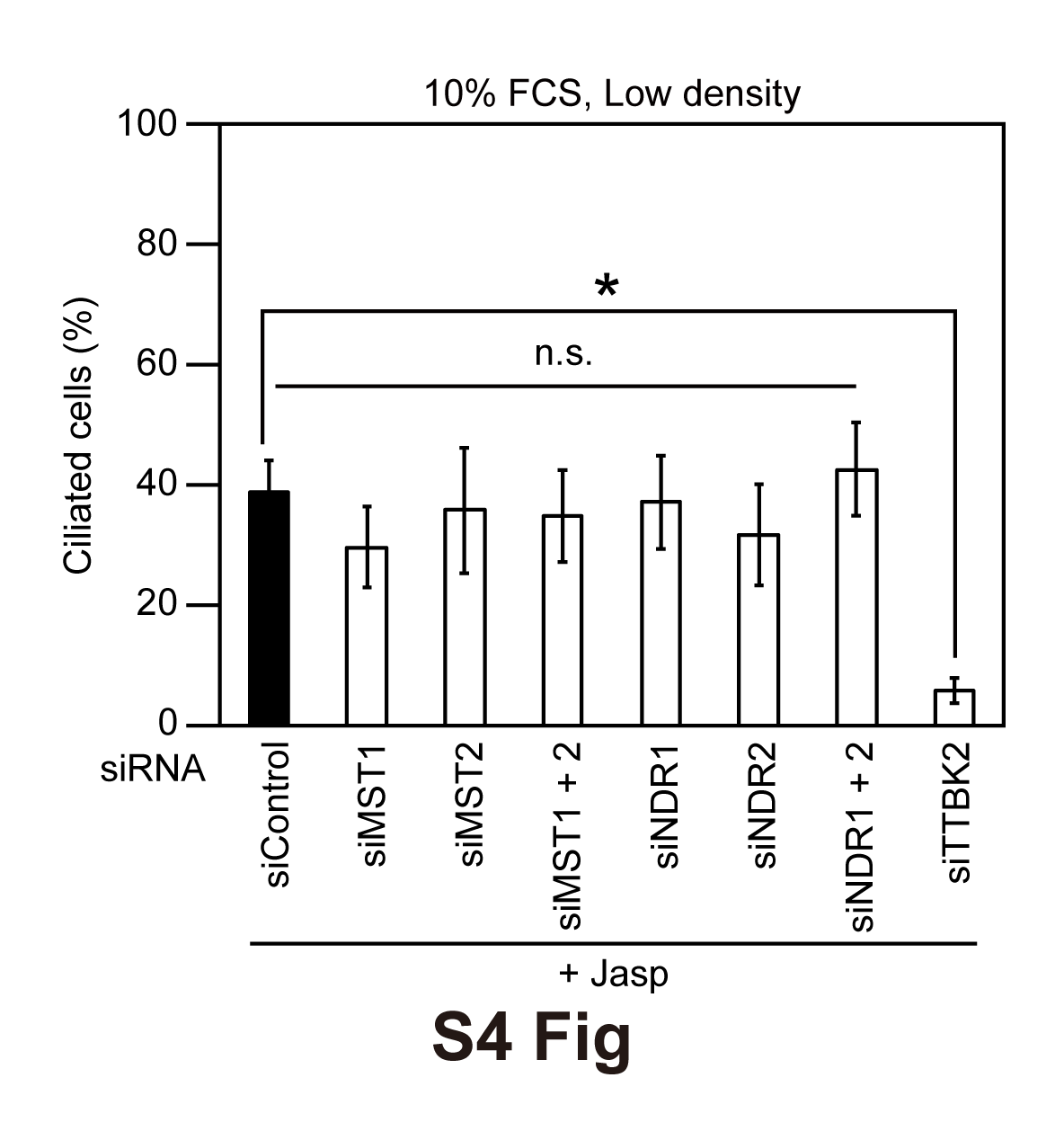

Supplement: S4 Fig — RPE1 cells were transfected with control siRNA or siRNAs targeting MST1, MST2, NDR1, NDR2, or TTBK2, as indicated; cultured at low density in serum-containing medium for 24 h; and then treated with 0.5 μM Jasp for 24 h. The percentage of ciliated cells was counted based on staining for Ac-tubulin and Arl13b. Data are means ± SEM from three independent experiments. *P < 0.05; n.s., not significant. (TIF) [file pone.0183030.s004.tif]

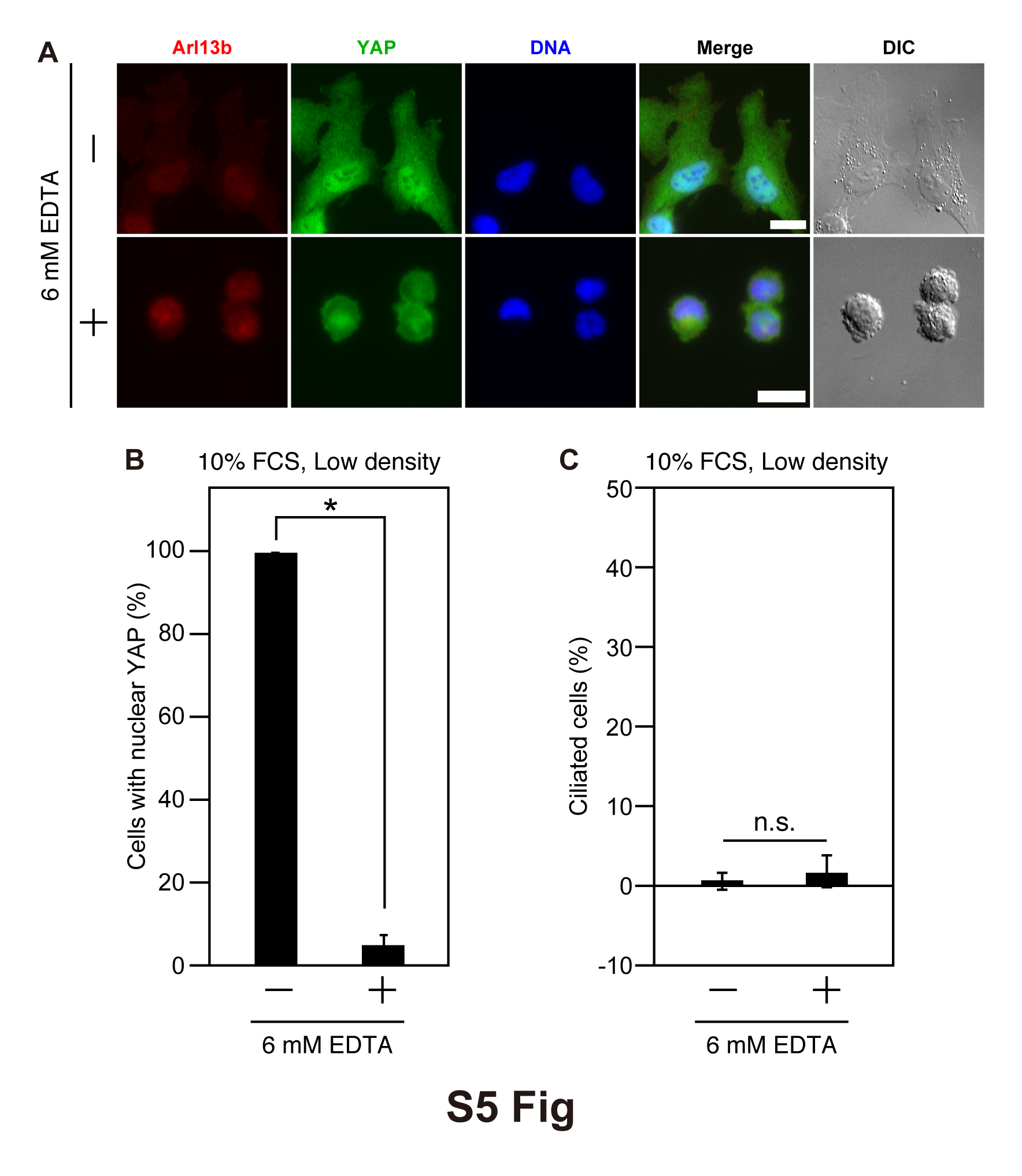

Supplement: S5 Fig — (A) EDTA treatment induces cell rounding and YAP translocation to the cytoplasm. RPE1 cells were cultured at low density; treated with 6 mM EDTA for 24 h; fixed and stained with anti-Arl13b (red) and anti-YAP (green) antibodies. DNA was stained with DAPI. DIC images are shown in the right panels. Scale bars, 20 μm. (B) Quantification of the effect of EDTA treatment on YAP localization. The percentage of cells with YAP localization in the nucleus was counted as in Fig 2C. (C) Quantification of the effect of EDTA treatment on ciliogenesis. The percentage of ciliated cells was counted based on staining of Arl13b, as shown in (A). In (B) and (C), data are means ± SEM from three independent experiments. n.s., not significant. (TIF) [file pone.0183030.s005.tif]
